# Sequencing of the Hepatitis C Virus: A Systematic Review

**DOI:** 10.1371/journal.pone.0067073

**Published:** 2013-06-27

**Authors:** Brendan Jacka, Francois Lamoury, Peter Simmonds, Gregory J. Dore, Jason Grebely, Tanya Applegate

**Affiliations:** 1 Viral Hepatitis Clinical Research Program, The Kirby Institute, University of New South Wales, Sydney, Australia; 2 Centre for Immunity, Infection and Evolution, University of Edinburgh, Edinburgh, United Kingdom; Kaohsiung Medical University Hospital, Kaohsiung Medical University, Taiwan

## Abstract

Since the identification of hepatitis C virus (HCV), viral sequencing has been important in understanding HCV classification, epidemiology, evolution, transmission clustering, treatment response and natural history. The length and diversity of the HCV genome has resulted in analysis of certain regions of the virus, however there has been little standardisation of protocols. This systematic review was undertaken to map the location and frequency of sequencing on the HCV genome in peer reviewed publications, with the aim to produce a database of sequencing primers and amplicons to inform future research. Medline and Scopus databases were searched for English language publications based on keyword/MeSH terms related to sequence analysis (9 terms) or HCV (3 terms), plus “primer” as a general search term. Exclusion criteria included non-HCV research, review articles, duplicate records, and incomplete description of HCV sequencing methods. The PCR primer locations of accepted publications were noted, and purpose of sequencing was determined. A total of 450 studies were accepted from the 2099 identified, with 629 HCV sequencing amplicons identified and mapped on the HCV genome. The most commonly sequenced region was the HVR-1 region, often utilised for studies of natural history, clustering/transmission, evolution and treatment response. Studies related to genotyping/classification or epidemiology of HCV genotype generally targeted the 5′UTR, Core and NS5B regions, while treatment response/resistance was assessed mainly in the NS3–NS5B region with emphasis on the Interferon sensitivity determining region (ISDR) region of NS5A. While the sequencing of HCV is generally constricted to certain regions of the HCV genome there is little consistency in the positioning of sequencing primers, with the exception of a few highly referenced manuscripts. This study demonstrates the heterogeneity of HCV sequencing, providing a comprehensive database of previously published primer sets to be utilised in future sequencing studies.

## Introduction

Given the difficulty in producing a continuous culture of the hepatitis C virus (HCV), investigations of HCV have been conducted mainly through the isolation and sequencing of the virus from infected patients. From this sequence data, a great deal has been learnt about the evolution of the virus within individuals, variability across geographic locations, and correlations between viral variation and clinical treatment response and disease progression. The methods to obtain this sequence data, however, have been largely diverse with little consensus in the sequencing methods applied within the field. Standardisation of methodologies is important in maintaining a high level of reliability in experimental output [1] and subsequent analysis [2], [3], however, it is more commonly undertaken in diagnostic testing than in the research setting.

Systematic reviews are commonly used in biomedical, epidemiology, public health and clinical research to provide an exhaustive summary of literature relevant to a specific research question, but are less common in the field of virology. Systematic and general reviews of published data related to sequencing of HCV have reported on genetic diversity [4], viral evolution [5], transmission of HCV [6] and predictors of treatment response or resistance [7], but there has been no review of the sequencing methodologies used for HCV.

Considering the wide application of sequencing in investigating HCV, our objective was to review HCV sequencing methodologies and to establish the variability of the regions sequenced. This systematic review provides a library of primers and regions for HCV sequencing, creating a valuable resource for other researchers in the field of HCV. From this, it may be possible to propose consensus regions that could be sequenced to increase the comparability and dissemination of HCV sequencing data. A systematic review of HCV sequencing is novel and similar methodologies could be applied to other viruses to enhance standardization and improve capacity for collaboration across disciplines.

## Results

### Study Selection

**Figure 1:**
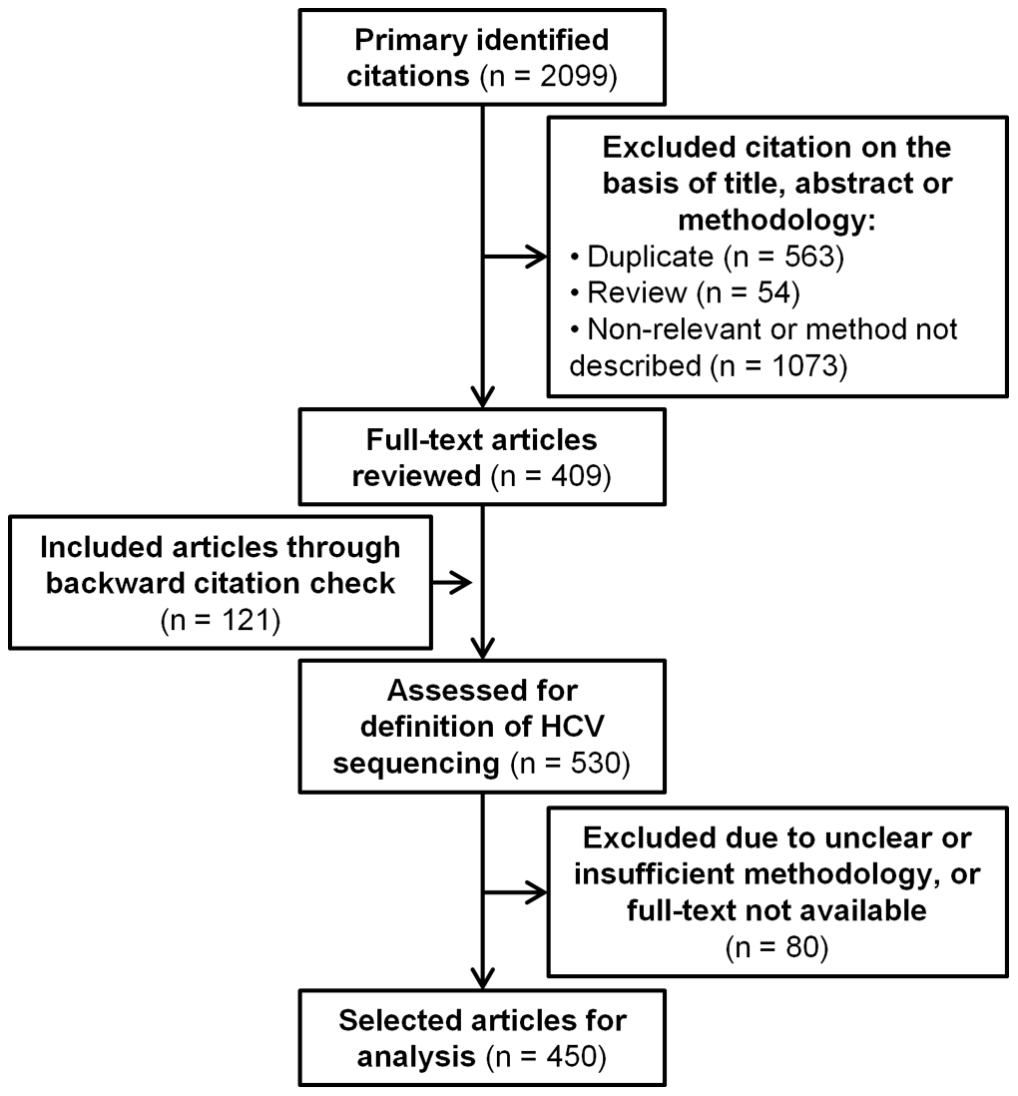
Flow diagram describing the review process and study selection.

A search of the Medline (by PubMed) and SCOPUS scientific databases identified 2099 publications with the selected keywords (Figure 1). From this a total of 1727 were not suitable for further analysis due to duplication of records (n = 563), being a review article (n = 54), or insufficient methodological description or irrelevance to HCV population sequencing (n = 1,046). This left a total of 407 publications to be assessed, which increased to 450 following identification of a further 43 publications through back-referencing. From these publications a total of 629 HCV population sequencing amplicons with positions relative to the H77 reference isolate [9] were identified.

Details of the selected publications including author list, title, date of publication, and journal of all publications accepted, selected primer sequence (where available) and 5′- and 3′-location of primers (according to the H77-reference sequence using Los Alamos National Laboratory *Quick Align* tool) are available in Table S1. A position-weighted matrix score representing the calculated nucleotide conservation in a 25 bp window as described by Qiu et al [10] is available for each primer in Table S1 and would allow for selection of primers with highly conserved nucleotide sequences.

### Study Characteristics

During analysis of the 450 publications the primary rationale for sequencing was categorised as clustering/transmission, epidemiology, evolution, genotyping/classification, natural history or treatment response/resistance. HCV RNA sequencing was most commonly performed for HCV genotyping/classification studies, with 37% of all identified sequencing amplicons (Table 1). In comparison, the remaining study groups each contributed between 9% and 17% of the amplicons identified. The median length of all HCV population sequencing amplicons was 384 bp (Lower and upper quartiles: 256, 592 bp), with treatment response/resistance studies having a median amplicon size considerably larger than other study groups. There were a number of sequencing amplicons that were full length or near full length, being approximately 9,646 bp long, with very few amplicons between 3,000 and 8,000 bp long (Figure S1-a).

**Table 1:** Amplicon size and positioning characteristics of the 608 sequencing amplicons identified in the systematic review of studies utilising HCV sequencing.

	Clustering/transmission	Epidemiology	Evolution	Genotyping/classification	Natural history	Treatment response/resistance	Combined
Number of publications (%)	42 (9.3)	41 (9.1)	79 (17.6)	170 (37.8)	58 (12.9)	70 (15.6)	450 (100)
Number of sequencing amplicons (%)	59 (9.4)	70 (11.1)	107 (17.0)	229 (36.4)	66 (10.5)	98 (15.6)	629 (100)
Median amplicon length (lower andupper quartiles)	384 (256, 571)	381 (271, 458)	393 (251, 625)	381 (251, 494)	378 (212, 710)	575 (357, 1312)	384 (256, 592)
Most commonly sequenced region	HVR1, NS5B	NS5B, 5′UTR-E1	HVR1; NS5B	5′UTR-Core; NS5B	HVR1	NS5A (ISDR)	HVR1, 5′UTR-Core, NS5B
Number of whole genome amplicons	0	0	8	17	7	5	37

Abbreviations: HVR1, Hypervariable region-1; NS5A, Non-structural gene 5a; NS5B, Non-structural gene 5b; 5′-UTR, 5′-untranslated region; E1. Envelope-1 gene; ISDR, Interferon Sensitivity Determining Region; 95% CI, 95% confidence interval.

### Description of HCV Population Sequencing

**Figure 2:**
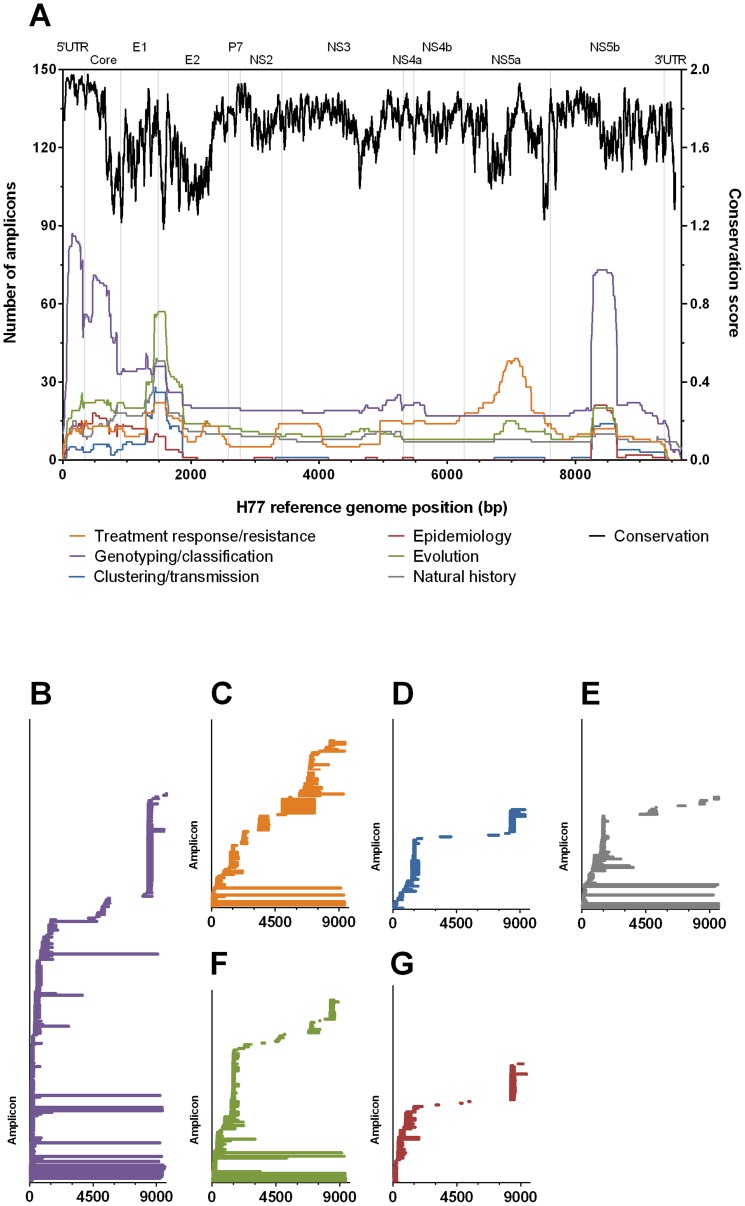
Positioning of combined and individual sequencing amplicons along the HCV genome. Sequencing amplicons identified in 434 peer-reviewed studies were categorised into study types, and their location mapped along the length of the HCV genome (A). A measure of genome diversity calculated by Qiu *et al* [10] illustrates the ranges of low and high genetic variation at regions frequently sequenced (e.g. 5′-untranslated region compared to 5′-region of E2 gene). When viewed individually, the sequencing amplicons show the heterogeneity of sequencing amplicon size and positioning for genotyping/classification (B), treatment response/resistance (C), clustering/transmission (D), natural history (E), evolution (F) and epidemiology (G) study types.

The regions of 5′UTR-core, hypervariable region-1 (HVR-1) and NS5B were the most commonly sequenced regions overall and within most of the different types of studies sequencing the HCV genome (Figure 2a). The exception was treatment response/resistance studies where a majority of population sequencing amplicons included the interferon sensitivity determining region (ISDR) of NS5A. At a number of regions on the HCV genome there was consensus on the positioning of the sequencing amplicon, as can be seen at the HVR-1 and NS5B regions. A position-weighted matrix score of viral diversity previously by Qiu *et al* [10], where a score of 0 and a score of 2 represents 0% and 100% similarity respectively, demonstrates the variability of HCV genome along its entire length (Black line in Figure 2a). When aligned to the positioning of HCV sequencing amplicons it can be seen that the highly conserved region of 5′UTR region and a moderately conserved segment in the NS5B gene were frequently sequenced for genotyping/classification. In contrast, the most genetically divergent portion of the HCV genome, being HVR-1, was rarely used in genotyping/classification but regularly sequenced in other analyses, particularly evolution and natural history.

As the positioning of sequencing/PCR primers would be constricted by the variation in the viral genome, it could be expected that there would be convergence in the design of these primers. Further assessment of individual sequencing amplicons reveals that while there is some degree of similarity in the positioning of PCR/sequencing primers, the majority of sequencing amplicons are unique in size or positioning (Figure 2b – g). It appears that it is more likely for sequencing amplicons to share sequencing primer locations in the HVR-1 and NS5B locations, but this may be influenced by the genetic variability, as seen by the Qiu *et al* [10] conservation score at these locations. There were a number of references to previously described sequencing primers throughout the literature, although it was not uncommon to see a 5′-primer from one method matched to a 3′-primer from a separate method (Table S1).

The number of published sequencing amplicons has decreased consistently over the past 15 years. There was a rapid rise in the number of publications between 1992 and 1996, peaking at 65 sequencing amplicons reported in that year (Figure S1-c). This was followed by a large decline in the number of reported amplicons to 1999, and a slower decline between 2000 and 2010 ending with 12 sequencing amplicons reported in 2011. The greatest contributor to the increase and subsequent decline was in the 1–500 bp sized sequencing amplicons, whereas publication of 501–1000 bp and greater than 1001 bp amplicons was relatively consistent over the time period (Figure S1-b).

## Discussion

In this systematic review of HCV population sequencing, there was considerable heterogeneity in the regions sequenced along the HCV genome. While there were examples of referencing to previous studies, the majority of studies described novel sequencing primers or amplicons. This heterogeneity in amplicon positioning may be a function of the high variability of the HCV genome or due to differing hypotheses tested by independent researchers. This study provides a comprehensive resource of HCV population sequencing primers and amplicons covering the entire HCV genome that may be used by future researchers across disciplines. The application of systematically reviewing sequencing data from other viruses with high genetic diversity (e.g. HIV) would enhance the standardisation of regions analysed for various study types and strengthen virological research in other fields.

There is clear consensus in the general positioning of sequencing amplicons in particular regions of HCV, such as 5′-UTR, HVR-1 and ISDR, however, the precise location of primers within these regions is not consistent. This diversity in primer design may affect the output and comparability of data due to differences in PCR/sequencing performance and bias [11], potentially leading to conflicting findings. Consensus guidelines for classification of new genotypes and subtypes [2], [3] proposed using either the entire genome or the Core/E1 and NS5B regions of HCV to identify novel strains, with particular mention of a segment of the NS5B gene first described by Enomoto *et al* [12]. As there are no guidelines for positioning of sequencing amplicons in studies not examining genotype/classification researchers have largely designed sequencing reactions independently, resulting in inconsistent positioning of HCV sequencing amplicons.

Across the 9.6 kb genome of HCV, there were selected regions that were more frequently sequenced than the rest of the genome: specifically the 5′-UTR, Core gene, HVR-1 and NS5B gene. A rationale for HCV sequencing assigned to each study revealed that the HVR-1 and NS5B regions were analysed in most of the study types, whereas the 5′-UTR and Core gene were predominately sequenced in studies undertaking HCV genotyping and/or classification. A segment of the non-structural 5A gene associated with interferon sensitivity (ISDR) was most often sequenced in studies assessing treatment response/resistance.

The development of directly acting antiviral agents (DAAs) specific to HCV with high efficacy has raised concerns about the potential for viral resistance mutations in the presence of these agents. Previous studies have identified and reported sequence [13] or amino acid [14] changes related to resistance phenotypes that lead to viral rebound during antiviral treatment. As next generation sequencing (NGS) and other novel technologies develop, population sequencing will be less frequently utilised in identifying these minor variants constituting a small percentage of the viral population in an infected individual.

The positioning of amplicons for HCV population sequencing is likely influenced by the high degree of variability of the virus genome, and the functions of various segments of the virus. Studies assessing response to standard interferon treatment may sequence the ISDR of NS5A as amino acid mutations in this region have been shown to correlate with treatment outcome in selected patients [15]. Determining the genetic classification of HCV may be performed using a number of regions (such as 5′-UTR, Core gene or NS5B gene) of the HCV genome that allow categorisation of isolates with related types [16], while the HVR-1 of E2 gene provides insight into the viral evolutionary responses at epitopes exposed to the host’s immune system [17].

Since HCV was initially described there has been a rapid increase in the number of studies utilising sequencing to analyse the virus. Following the release of a proposed nomenclature for HCV genotyping by Simmonds *et al* [2] there was a peak in the number of sequencing reactions in 1996, followed by a decline until 1999 and a steady number since. This decrease in reporting of HCV sequencing may have resulted from the production of commercial HCV genotyping methods, such as Inno-LiPA HCV Genotype 1 in 1996 [18]. The greatest reduction in reported sequencing reactions was in the less than 500 bp category, with relatively stable numbers of larger sized amplicons, potentially reflecting the shift away from HCV sequencing as a genotyping tool towards biological and functional analysis of the HCV virus requiring longer amplicons.

This study has several limitations. The selection of studies for this study included a search of PubMed and Scopus using HCV or sequencing-specific keywords, resulting in more than 2000 studies to review, of which over 75% were excluded based on evaluation of content. The remaining studies were sorted into categories according to the aim of the study. These processes were undertaken manually and may have contributed to errors in the selection and characterisation of the dataset. Studies relevant to this systematic review may not have been identified if they did not include the keyword terms used in the search, potentially resulting in an underestimation of the overall number of studies describing HCV sequencing. However, a large number of search terms were used and additional studies were included through back-referencing.

This study provides the first reported review of population sequencing methodology used in the HCV field, and provides a comprehensive list of the population sequencing primers from these studies. The review methodology in this study provides a platform for further review and standardization of sequencing methods undertaken in areas of research where high genetic diversity may impact on the comparability of results, such as HIV, hepatitis B virus and other infectious diseases. From the review of the HCV sequencing amplicons, it was noted that there was great heterogeneity in the positioning of population sequencing amplicons in studies performed to date. This database of HCV sequencing primers is a valuable resource for researchers in the field of HCV that could be used to increase the comparability and sharing of HCV sequencing data. The extensive heterogeneity of the sequencing regions suggests potential for some standardisation of regions analysed for each study type to strengthen future HCV virological research.

## Methods

This systematic review was written according to the Preferred Reporting Items for Systematic Reviews and Meta-Analyses (PRISMA) statement [8]. Given this statement was originally developed for studies evaluating healthcare interventions, components of this statement have been customized to fit the scope of the current systematic review (Table S2).

### Eligibility Criteria

All primary research papers that reported sequencing of the HCV genome were included if they were:

- published in English; and
- conducted on human individuals.

### Information Sources

A literature search of MEDLINE (PubMed) and SCOPUS was performed on 11^th^ December, 2011, covering all studies published. The list of eligible articles found during the initial search was hand searched for reference to sequencing of the HCV genome and backward citation checks were carried out to identify further potentially relevant studies. Review articles were not included in this analysis.

### Search Strategies

Medline Search (MeSH) terms were used to search for studies relating to HCV ("Hepacivirus" OR "Hepatitis C Antibodies" OR "Hepatitis C") and sequencing ("Cluster analysis" OR "Molecular sequence data" OR "Base sequence" OR "Sequence Analysis" OR "Molecular Epidemiology" OR "Sequence Homology" OR "Sequence alignment" OR "Drug resistance") from these two databases. The general search term “primer” was applied to identify studies that utilised sequencing as a method as this term is strongly related with PCR and sequencing.

### Study Selection

The lists of studies from each search database were combined and ineligible articles removed based on the following criteria: duplicate reference, review article, abstract or title accessible only, description of next generation sequencing (NGS) without population sequencing, or not relevant/insufficient method description. Studies were deemed eligible for this analysis if HCV population sequencing with PCR/sequencing primer design or location was described in the methodology section. Publications utilising sequencing to confirm the accuracy of PCR products only were excluded from the analysis, however those that performed sequencing of a different region to validate a new method were included.

### Data Collection and Analysis

The genome location of HCV PCR/sequencing primers was recorded for eligible studies, or back references noted where methodology was described by other studies. For studies that described a nested PCR protocol or specific sequencing primer the innermost primer was used for the amplicon analysis. The innermost primer pair was also selected where there were two or more primer pairs covered an identical region, for example when genotype-specific primer pairs were described. Where a sequencing region was derived from multiple overlapping sequencing amplicons the outermost primers were selected at the 5′- and 3′-primers respectively.

Eligible studies were categorised based on primary study focus as either 1) Genotyping/classification, 2) Clustering/transmission, 3) Evolution, 4) Treatment response/resistance, 5) Epidemiology, or 6) Natural history.

## Supporting Information

Table S1 **Database of HCV sequencing amplicons.** Including author list, title, date of publication, and journal of accepted publications, selected primer sequence (where available) and 5′- and 3′-location of primers.
(XLSX)

Table S2 **PRISMA checklist.**
(DOC)

Figure S1 **Distribution of sequencing amplicons according to size and year of publication.** The size distribution of HCV amplicons (A) shows a bias towards products smaller than 1000 bp in length, with fewer than 10% of amplicons being larger than this. There is a rapid increase in the number of publications until a peak in 1996. The decrease in reported sequencing amplicons may be attributed to fewer amplicons sized 1–500 bp (B) and a sharp drop in sequencing for genotyping/classification from 1998 onwards. There were fluctuations in the numbers of reported sequencing amplicons for all study types over the time period (C).
(TIF)
