# Supplementary figures and images for: Sequencing of the Hepatitis C Virus: A Systematic Review
Source: PLoS One. 2013 Jun 27;8(6):e67073. doi: 10.1371/journal.pone.0067073 (PMC3694929; doi:10.1371/journal.pone.0067073)

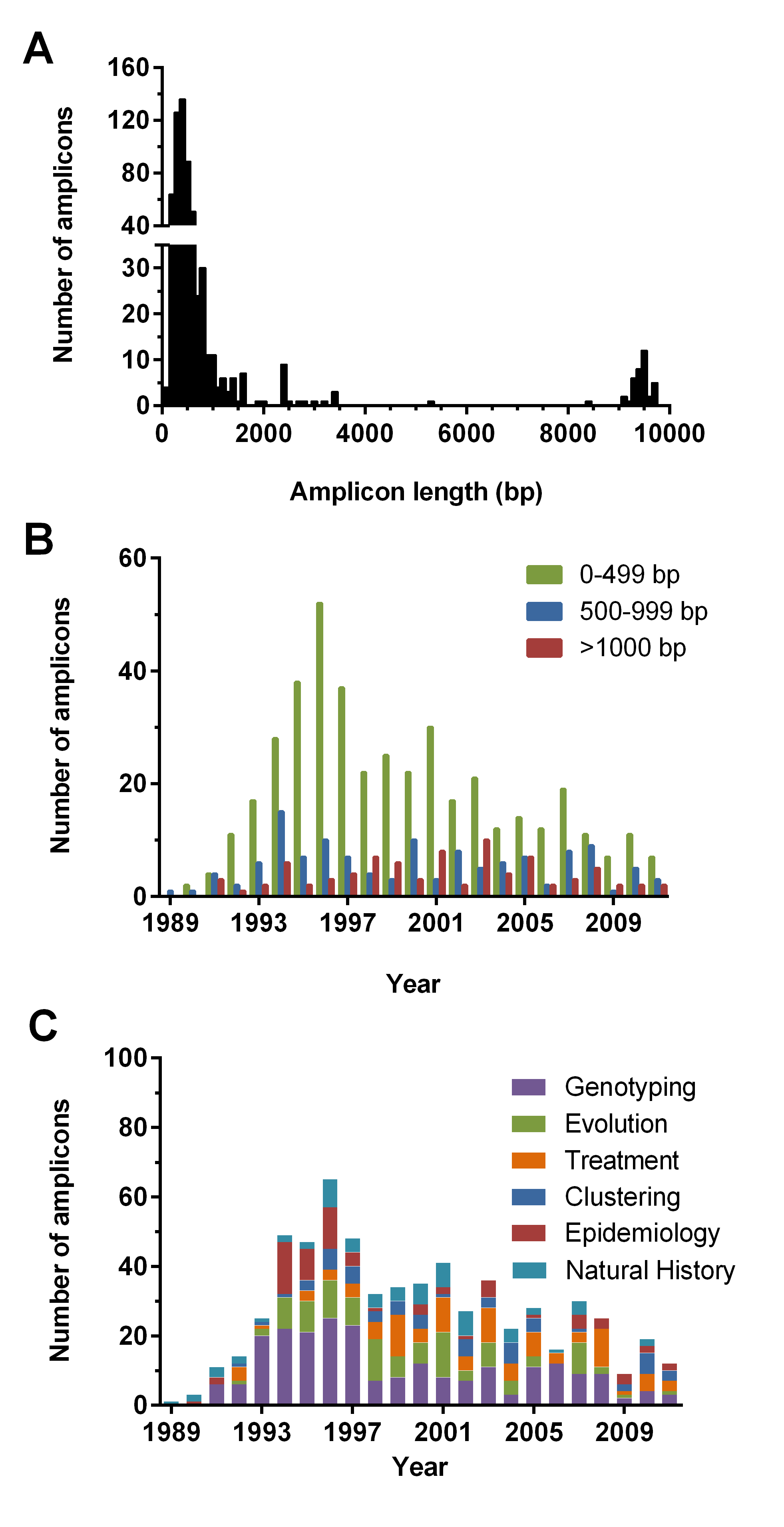

Supplement: Figure S1 — Distribution of sequencing amplicons according to size and year of publication. The size distribution of HCV amplicons (A) shows a bias towards products smaller than 1000 bp in length, with fewer than 10% of amplicons being larger than this. There is a rapid increase in the number of publications until a peak in 1996. The decrease in reported sequencing amplicons may be attributed to fewer amplicons sized 1–500 bp (B) and a sharp drop in sequencing for genotyping/classification from 1998 onwards. There were fluctuations in the numbers of reported sequencing amplicons for all study types over the time period (C). (TIF) [file pone.0067073.s003.tif]
